# Anemia Secondary to Gastric Antral Vascular Ectasia Successfully Treated With Antral Gastrectomy

**DOI:** 10.7759/cureus.69663

**Published:** 2024-09-18

**Authors:** Francisco Girão de Caires, Mafalda Nunes, Filipe Damiao, Isabel Dionísio, Adelaide Gomes da Costa

**Affiliations:** 1 General Surgery, Unidade Local de Saúde do Oeste, Caldas da Rainha, PRT; 2 Gastroenterology, Unidade Local de Saúde do Oeste, Caldas da Rainha, PRT

**Keywords:** antrectomy, argon plasma coagulation, endoscopic band ligation, endoscopy, gastrectomy, gastric antral vascular ectasia, gastrointestinal bleeding, surgery, watermelon stomach

## Abstract

Gastric antral vascular ectasia (GAVE) is a rare but significant cause of chronic gastrointestinal bleeding and anemia, particularly in elderly patients. We report the case of a 75-year-old female who presented with severe anemia secondary to GAVE. Despite multiple endoscopic interventions with argon plasma coagulation (APC) treatments and endoscopic band ligation (EBL), the patient’s condition persisted, necessitating an antral gastrectomy with intraoperative endoscopy to delineate the proximal resection margin. Postoperative outcomes were favorable, with no recurrence of anemia or gastrointestinal bleeding observed during follow-up.

## Introduction

Gastric antral vascular ectasia (GAVE), often referred to as “watermelon stomach” due to its endoscopic appearance, is a rare cause of chronic gastrointestinal blood loss leading to anemia [1]. First described in 1953 by Rider et al., and later more comprehensively by Jabbari in 1984, GAVE is characterized by dilated capillaries and fibrin thrombi within the gastric antrum's mucosa, leading to chronic blood loss [1].

GAVE has a strong association with systemic diseases, particularly chronic liver disease and cirrhosis, where it is seen in 30% of patients with cirrhosis [2]. It has also been linked to autoimmune diseases such as systemic sclerosis, as well as chronic renal failure, among others [2]. The pathophysiology of GAVE remains incompletely understood, but it is thought to involve mechanical stress on the gastric mucosa, alterations in mucosal blood flow, and hormonal dysregulation, particularly involving gastrin and vasoactive peptides.

GAVE predominantly affects older adults and can be associated with significant morbidity [2]. While endoscopic treatments such as argon plasma coagulation (APC) and endoscopic band ligation (EBL) are commonly employed, they may not be effective in all cases. Surgical intervention remains an option for refractory cases [3]. This report presents a case of GAVE in a 75-year-old female, detailing her treatment course and eventual surgical management.

## Case presentation

A 75-year-old female with a history of chronic anemia presented with symptoms of fatigue. Her medical history included hypertension, osteoarthritis, and gallstone disease with no significant family history of gastrointestinal disorders.

**Figure 1:**
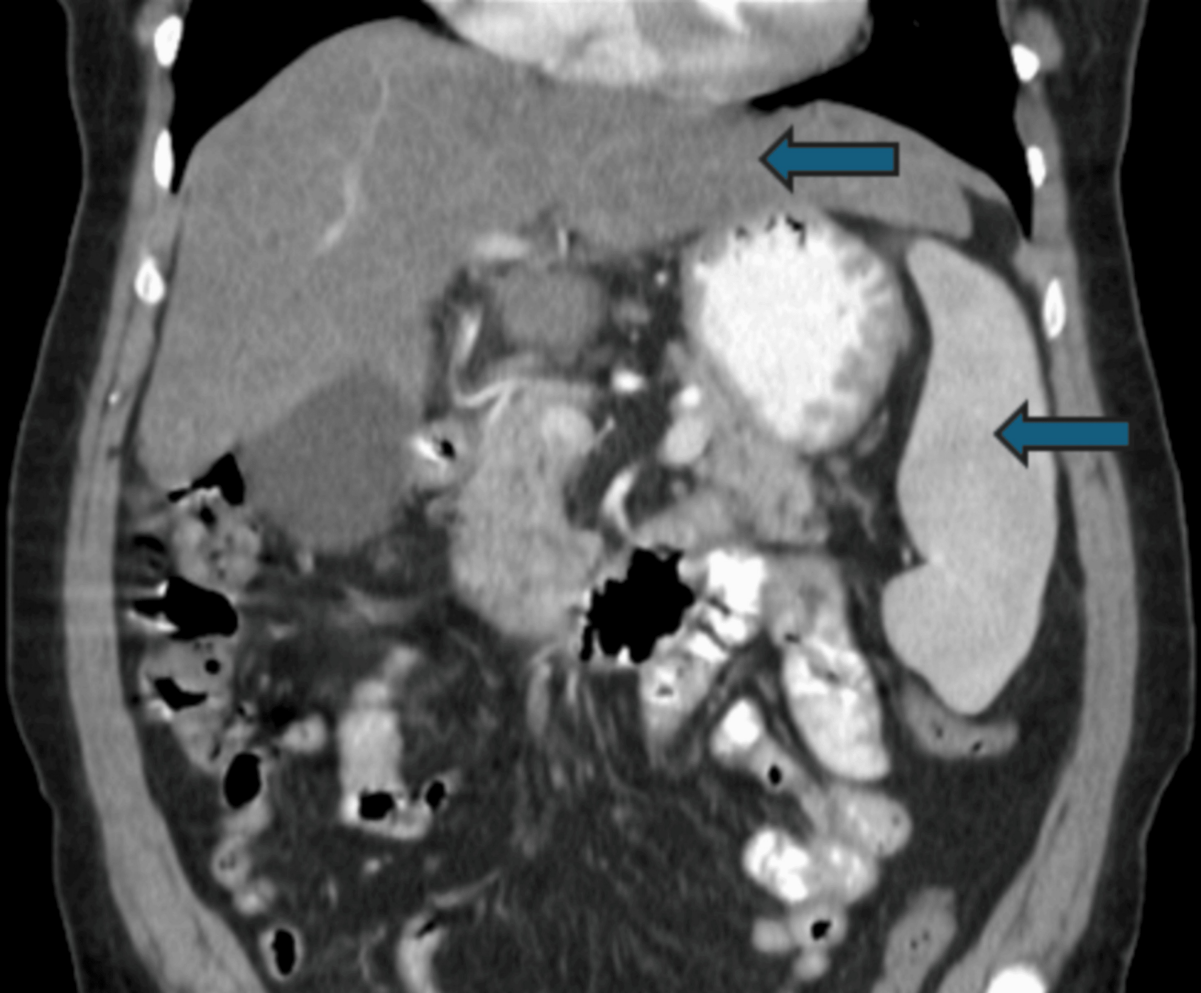
CT scan revealing hepatosplenomegaly (blue arrows)

The patient exhibited pallor and signs of iron deficiency anemia. Initial laboratory tests revealed a hemoglobin level of 6.7 g/dL, with microcytic hypochromic indices. The computed tomography (CT) scan revealed hepatosplenomegaly and signs consistent with chronic liver disease (Figure 1).

**Figure 2:**
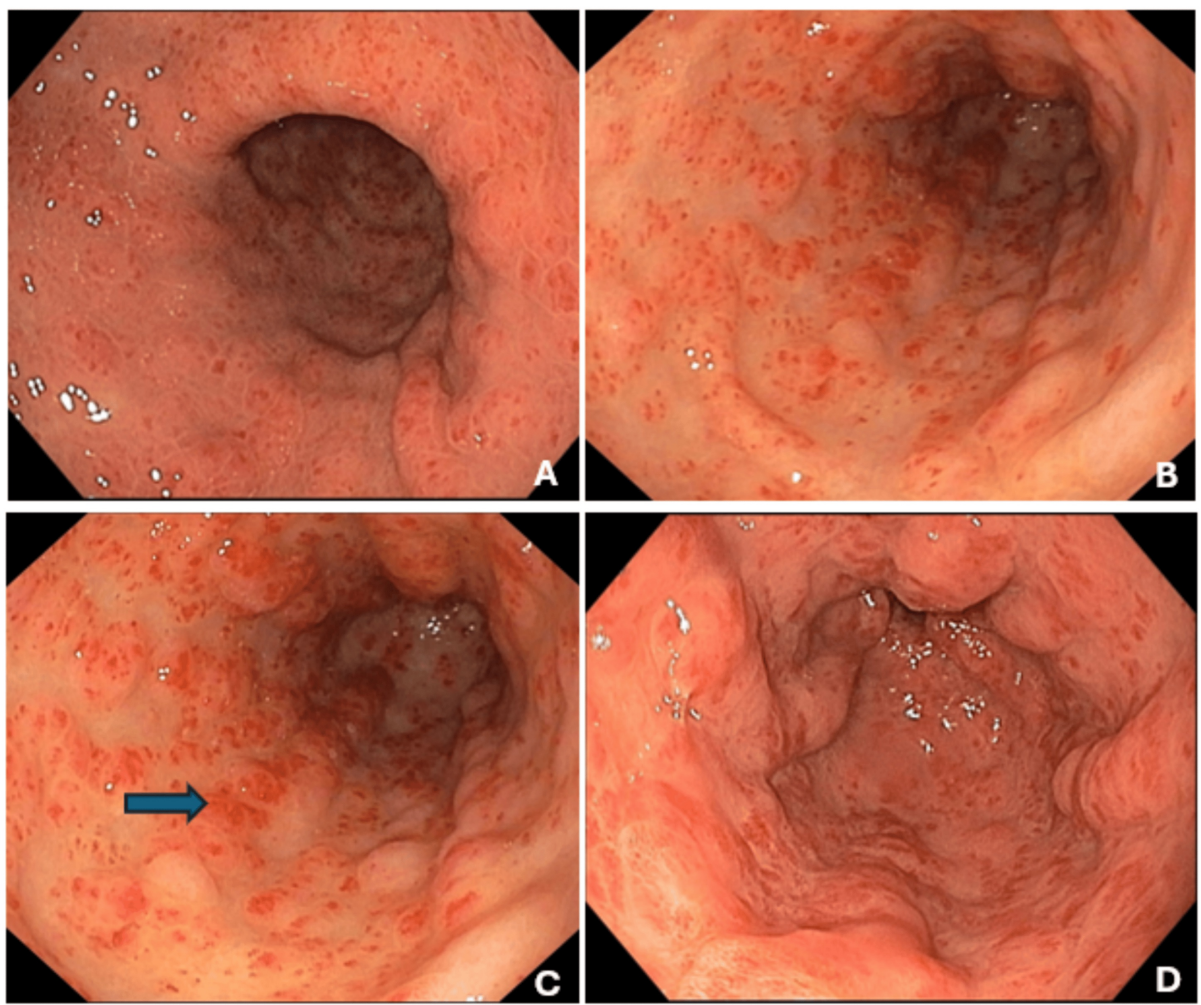
Upper gastrointestinal endoscopy (A-D) Proximal to distal gastric antrum; blue arrow: vascular ectasia

Upper gastrointestinal endoscopy demonstrated characteristic GAVE with multiple linear erythematous streaks radiating from the pylorus. Biopsies revealed the presence of superficial hyperplastic antral mucosa, capillary ectasia with thrombosis and fibromuscular hypertrophy of the lamina propria confirming the diagnosis (Figures 2A-2D).

**Figure 3:**
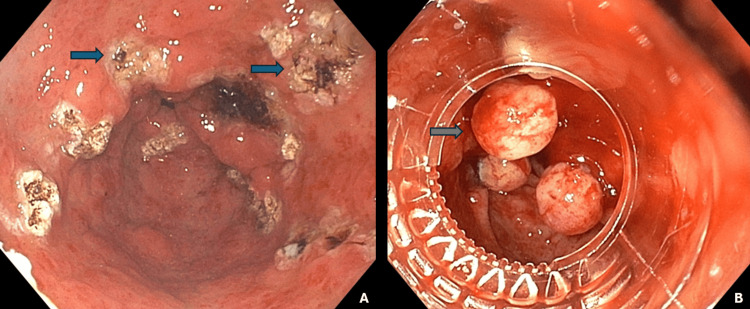
Endoscopic treatments (A) Fulguration scars (blue arrows) after argon plasma coagulation. (B) Elastic banding ligation (grey arrow).

Given the chronic and recurrent nature of her anemia, initial management included multiple sessions of APC (Figures 3A, 3B). Despite undergoing several endoscopic APC and EBL treatments, the patient continued to experience recurrent anemia requiring frequent blood transfusions. Given the persistence of symptoms and the underlying liver disease, a multidisciplinary team decided to pursue surgical intervention.

**Figure 4:**
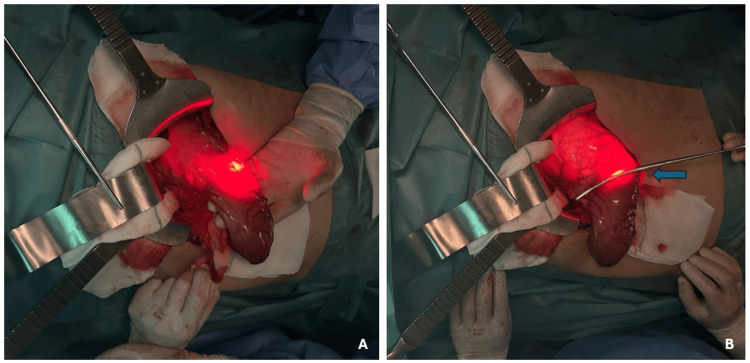
(A, B) Intraoperative upper gastrointestinal endoscopy and determination of the line of section of the gastric antrum (blue arrow)

The patient underwent a Roux-en-Y antral gastrectomy with intraoperative endoscopy to determine the precise proximal margin of resection. A cholecystectomy was also performed. The procedure was uneventful, and intraoperative endoscopy confirmed the complete excision of the affected gastric antrum (Figures 4A, 4B).

**Figure 5:**
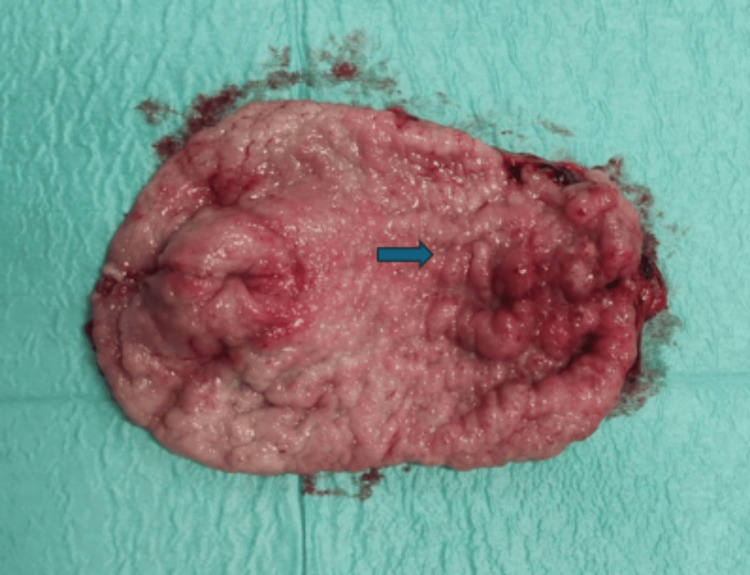
Antrectomy specimen showing the complete excision of the affected gastric antrum (blue arrow)

The complete GAVE excision was evidenced by the macroscopic observation of the excised specimen (Figure 5).

Postoperatively, the patient had an uneventful recovery. Her hemoglobin levels stabilized without the need for further blood transfusions. Follow-up endoscopy at six months showed no recurrence of GAVE or gastrointestinal bleeding. The patient reported significant improvement in her symptoms and quality of life.

## Discussion

GAVE is a rare but significant cause of chronic gastrointestinal bleeding and anemia, predominantly affecting older adults [1]. It is characterized by the presence of dilated small blood vessels in the antral part of the stomach, leading to chronic blood loss and resultant anemia. The etiology of GAVE is not well understood, but it is frequently associated with systemic conditions such as liver cirrhosis, systemic sclerosis, and chronic renal failure [2].

The pathogenesis of GAVE involves abnormal vascular proliferation and fibromuscular hyperplasia in the gastric antrum. The distinctive endoscopic appearance, which resembles the stripes of a watermelon, helps in the diagnosis. Biopsies are often performed to confirm the diagnosis and rule out other conditions such as portal hypertensive gastropathy [4]. The patient's CT findings of hepatosplenomegaly and chronic liver disease are noteworthy, as chronic liver disease is a known association with GAVE and can complicate the management of the condition [5].

Endoscopic treatment is typically the first line of management for GAVE. APC and endoscopic banding ligation (EBL) are commonly used due to their effectiveness in coagulating the superficial blood vessels, thus reducing bleeding. Studies have shown that APC and EBL can achieve hemostasis in a significant proportion of patients; however, recurrence is common, necessitating repeated sessions [3].

In this case, despite multiple sessions of endoscopic treatments, the patient continued to experience significant anemia requiring frequent blood transfusions. This highlights a major limitation of endoscopic treatments in some patients with GAVE, where the response to APC and EBL can be inadequate, and recurrent bleeding remains a challenge [6].

For refractory cases of GAVE, surgical intervention can be considered. Antral gastrectomy, which involves the resection of the affected antral portion of the stomach, is effective in controlling bleeding in patients unresponsive to endoscopic treatment. The use of intraoperative endoscopy is crucial in ensuring complete resection of the affected area, thereby minimizing the risk of recurrence [7].

In this patient, an antral gastrectomy was performed with intraoperative endoscopy to determine the proximal margin of resection. This approach was successful, as evidenced by the stabilization of her hemoglobin levels postoperatively and the absence of recurrent gastrointestinal bleeding during follow-up. The decision to proceed with surgery was driven by the refractory nature of her condition and the failure of endoscopic management to control her bleeding adequately.

This case underscores the importance of a multidisciplinary approach in managing complex cases of GAVE. While endoscopic treatments remain the mainstay for most patients, surgical options should be considered for those with refractory disease. The successful outcome in this patient demonstrates that antral gastrectomy, when combined with intraoperative endoscopy, can provide a definitive cure for GAVE, improving the patient’s quality of life and eliminating the need for ongoing blood transfusions [8].

Further research is needed to identify predictors of poor response to endoscopic treatment in patients with GAVE. Additionally, the development of new endoscopic techniques or adjunctive therapies may improve outcomes for patients who are not candidates for surgery. Understanding the underlying mechanisms of GAVE and its association with systemic diseases may also lead to better-targeted therapies [9,10].

## Conclusions

This case report outlines the management of a 69-year-old woman with anemia secondary to GAVE, compounded by chronic liver disease and hepatosplenomegaly. Despite multiple sessions of endoscopic treatments, the patient continued to experience recurrent gastrointestinal bleeding and severe anemia, necessitating frequent blood transfusions. Given the refractory nature of her condition, an antral gastrectomy with intraoperative endoscopy to define the proximal margin of resection was performed, leading to a favorable outcome with no recurrence of bleeding or anemia postoperatively.

GAVE should be considered in the differential diagnosis of elderly patients presenting with chronic anemia and gastrointestinal bleeding. While endoscopic treatments are effective for many patients, surgical interventions, such as antral gastrectomy, may be necessary for refractory cases. This case highlights the successful use of antral gastrectomy with intraoperative endoscopy to achieve definitive control of GAVE-related bleeding.
